# Quality assessment of cardiac magnetic resonance myocardial scar imaging prior to ventricular arrhythmia ablation

**DOI:** 10.1007/s10554-022-02734-5

**Published:** 2022-11-04

**Authors:** Rushil Shah, Apurva Sharma, Fabrizio Assis, Henrique Doria De Vasconcellos, Navya Alugubelli, Pallavi Pandey, Tauseef Akhtar, Alessio Gasperetti, Shijie Zhou, Henry Halperin, Stefan L. Zimmerman, Harikrishna Tandri, Aravindan Kolandaivelu

**Affiliations:** 1 Division of Cardiology, Department of Medicine, Johns Hopkins University School of Medicine, Carnegie 528, 600 N. Wolfe St, Baltimore, MD 21287 USA; 2 Department of Radiology and Radiological Science, Johns Hopkins University School of Medicine, Baltimore, MD USA; 3 Division of Cardiology, Department of Medicine, Creighton University, Omaha, NE USA; 4 Department of Chemical, Paper and Biomedical Engineering, Miami University, Oxford, OH USA; 5 Department of Electrical and Chemical Engineering, Miami University, Oxford, OH USA

**Keywords:** Cardiac magnetic resonance imaging, Late gadolinium enhancement (LGE), Arrythmia, Ventricular tachycardia (VT), Implantable cardioverter defibrillator (ICD), Motion artifact, Catheter ablation

## Abstract

High-resolution scar characterization using late gadolinium enhancement cardiac magnetic resonance imaging (LGE-CMR) is useful for guiding ventricular arrhythmia (VA) treatment. However, imaging study quality may be degraded by breath-holding difficulties, arrhythmias, and implantable cardioverter-defibrillators (ICDs). We evaluated the effect of image quality on left ventricle (LV) base to apex scar interpretation in pre-VA ablation LGE-CMR. 43 consecutive patients referred for VA ablation underwent gradient-recalled-echo LGE-CMR. In ICD patients (n = 24), wide-bandwidth inversion-recovery suppressed ICD artifacts. In non-ICD patients**,** single-shot steady-state free-precession LGE-CMR could also be performed to reduce respiratory motion/arrhythmia artifacts. Study quality was assessed for adequate/limited scar interpretation due to cardiac/respiratory motion artifacts, ICD-related artifacts, and image contrast. 28% of non-ICD patients had studies where image quality limited scar interpretation in at least one image compared to 71% of ICD patient studies (p = 0.012). A median of five image slices had limited quality per ICD patient study, compared to 0 images per non-ICD patient study. Poorer quality in ICD patients was largely due to motion-related artifacts (54% ICD vs 6% non-ICD studies, p = 0.001) as well as ICD-related image artifacts (25% of studies). In VA ablation patients with ICDs, conventional CMR protocols frequently have image slices with limited scar interpretation, which can limit whole-heart scar assessment. Motion artifacts contribute to suboptimal image quality, particularly in ICD patients. Improved methods for motion and ICD artifact suppression may better delineate high-resolution LGE scar features of interest for guiding VA ablation.

**Supplementary Information:**

The online version contains supplementary material available at 10.1007/s10554-022-02734-5.

## Introduction

Cardiac magnetic resonance imaging (CMR) is the gold standard method for characterizing myocardial scar because of its superior soft tissue contrast to modalities such as X-ray computed tomography (CT) and ultrasound and higher resolution compared to nuclear scans such as single photon emission computed tomography (SPECT) and positron emission tomography (PET) [1]. Because of the central role of myocardial scar in ventricular tachycardia pathophysiology, CMR is of increasing interest in ventricular arrhythmia (VA) risk prediction as well as for guiding catheter ablation.

Catheter ablation is often used to treat VA that is refractory to anti-arrhythmic drugs [2]. In patients with structural heart disease, visualization of myocardial scar is a pre-requisite step to performing VA ablation. Scar visualization has been conventionally performed by manipulating a catheter across the surface of the myocardium and making invasive measurements. This process, termed electro-anatomic mapping (EAM) can be time consuming and has limited ability to detect arrhythmogenic tissue deeper within the myocardium.

CMR scar information can be imported into a three-dimensional (3D) EAM system to compliment and better characterize complex 3D myocardial scar anatomy that leads to ventricular tachycardia (VT). Late Gadolinium Enhancement (LGE) CMR imaging can detect the regional and transmural location of myocardial scar and help direct invasive catheter manipulation and ablation [3]. In addition, higher-resolution CMR scar features, such as heterogenous tissue channels (HTCs), could more specifically target ablation [4]. Ablation of CMR identified targets has been associated with lower inducibility rates and improved VT-free survival [5].

However, difficulty in breath-holding and presence of arrhythmias during image acquisition can degrade CMR image quality and compromise scar assessment in patients with VT [6]. In addition, many VT ablation patients with structural heart disease have implantable cardiac defibrillators (ICD). Though initial safety concerns of performing CMR in ICD patients have been mitigated, ferromagnetic components within ICDs distort the MRI scanner magnetic field leading to image artifacts that can limit interpretation of image pathology [7, 8]. This study retrospectively evaluated the impact of motion and ICDs on scar imaging quality and clinical interpretation in a group of patients who underwent CMR prior to VT ablation.

## Methods

### Study population

Written informed consent for this retrospective image review was obtained before the patient’s ablation procedure as part of consent to participate in the Johns Hopkins VT ablation registry. The registry study protocol was reviewed and approved by the Johns Hopkins Institutional Review Board. All pre-VA ablation CMR studies during the period from September 2015 to January 2020 were evaluated, following availability of the wide-bandwidth inversion-recovery (wbIR) technique that improves LGE-CME scar assessment in patients with ICDs [9].

### CMR image acquisition

**Fig. 1:**
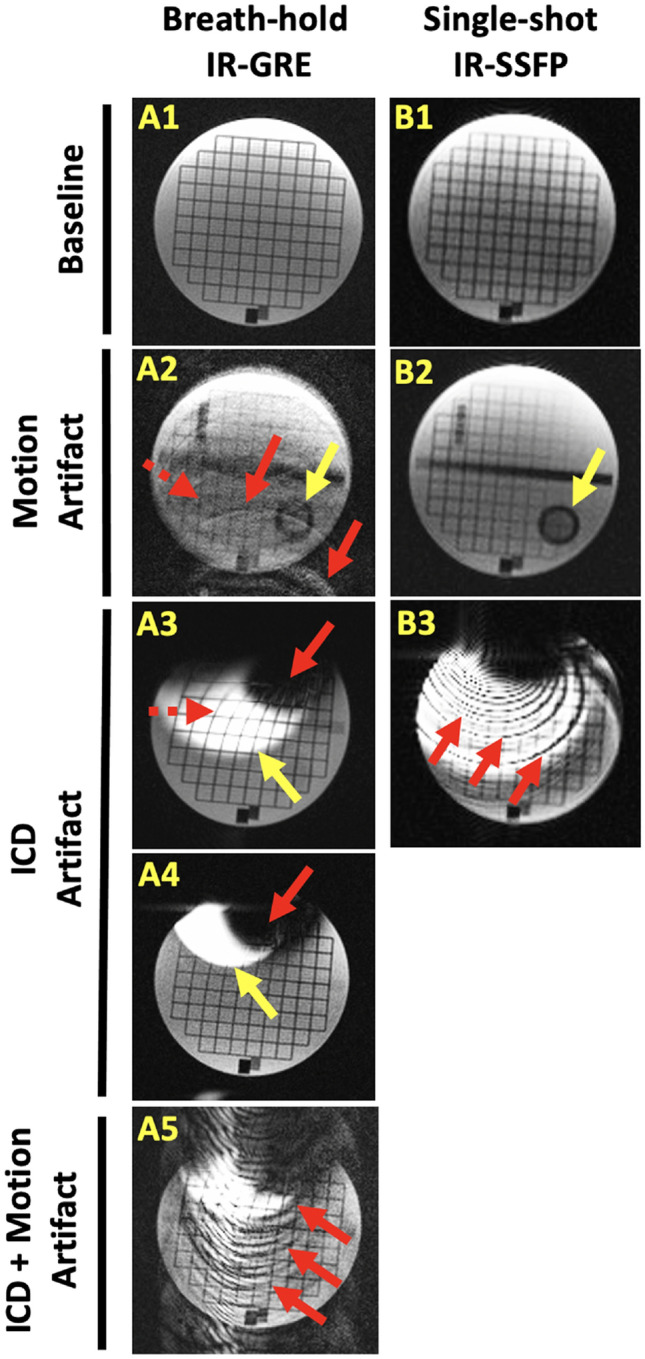
Illustration of motion and ICD artifacts for conventional LGE MRI techniques used for scar imaging. **A1**–**5** Cardiac gated, breath-hold inversion-recovery (IR) gradient recalled echo (GRE) imaging collected with simulated heart rate 75 bpm. **A1** image without motion is sharper than **A2** with 2 cm, 3 Hz motion simulating poor breath-holding. Motion causes ghosting artifacts (e.g., red arrows), blurring (e.g., dashed red arrow), and appearance of structures that fall outside the desired image plane (e.g., yellow arrow). **A3** placement of an ICD over the stationary object introduces different image artifacts caused by ferromagnetic ICD components. Hyperintensity occurs in areas that fall outside the bandwidth of the IR-pulse (yellow arrow). Distortion of the grid (dashed red arrow) and signal void (red arrow) are seen closer to the ICD, **A4** Wider-bandwidth IR reduces hyperintensity artifact (yellow arrow). Higher receiver bandwidth additionally decreases image distortion closer to the ICD. **A5** In the presence of an ICD, conspicuous ghosting artifacts are seen during 2 cm simulated respiratory motion (red arrows). **B1**–**3** Single-shot IR balanced steady state-free precession (SSFP) imaging. **B1** image without motion has lower image resolution and increased image noise compared to breath-hold imaging. **B2** However, with 2 cm simulated respiratory motion, images have less blurring and ghosting compared to the breath-hold imaging. Still, objects outside the desired image plane can be seen (yellow arrow). B3) SSFP imaging is less useful for imaging subjects with ICDs due to significant banding artifacts

CMR was performed on 1.5 T MRI scanners (Aera and Avanto, Siemens Medical Systems). Thirteen to fifteen short-axis LGE CMR image planes were typically acquired, spanning the left ventricle (LV) base to apex. Breath-hold, ECG-gated LGE CMR was performed using conventional phase sensitive inversion recovery (PSIR) gradient recalled echo imaging (bh-LGE) with one image plane per breath-hold [10]. In patients without ICDs, single-shot steady-state free-precession PSIR late gadolinium enhancement imaging (ss-SSFP-LGE) was also performed to reduce possible respiratory and cardiac motion artifacts (Fig. 1A2 vs. B2) [10]. In patients with ICDs, bh-LGE was performed using wide-bandwidth inversion recovery (wbIR) to suppress hyperintensity artifacts that could otherwise affect scar interpretation (Fig. 1A3 vs. A4) [9]. Ss-SSFP-LGE imaging was not performed in ICD patients due to prohibitive artifact (Fig. 1B3).

### CMR image interpretation

LGE study quality was determined from base to apex short-axis images of the LV. Study quality was evaluated in terms of motion artifacts and ICD artifacts (Fig. 1). Image contrast quality was assessed by whether scar or absence of scar could be interpreted above image noise, apart from artifacts due to motion and ICDs. Overall quality was determined considering motion, ICD artifacts, and image contrast quality. Analysis focused on short-axis imaging, which was performed more consistently between patients than long-axis imaging. Quality of each image was graded as “acceptable”, ie. artifacts not affecting image interpretation, or “limited”, ie. artifacts that could affect image interpretation. In cases where imaging of the same slice location was repeated, the best quality score was used. Study quality was assigned the poorest score of any short-axis image for each quality parameter (e.g., motion artifact). For non-ICD participants, in whom bh-LGE and ss-SSFP-LGE imaging were both performed, the better of the two LGE studies was used for overall quality scoring. Agreement of scar presence/location was evaluated between the official radiology read and a CMR experienced electrophysiologist. Agreement was assessed for “clear scar” as well as “clear and possible scar”. Clear scar was defined as scar that was felt clearly detectable above the image noise/artifact level. “Possible scar” was defined as scar that was considered less definitively detected above the image noise/artifact level. Study assessments were reviewed and agreed upon by a second experienced CMR reader.

### Statistical analysis

Continuous variables were summarized as median and interquartile range [IQR] and compared using Mann–Whitney *U* Test with 95% confidence intervals. Two tailed p values < 0.05 were considered statistically significant. Categorical variables were listed as numbers or proportions and differences tested using Fisher’s exact test. Univariate linear regression identified demographic variables that were significantly associated with “limited” overall study quality. Multivariate regression was performed for significantly associated variables (p < 0.15) to identify independent predictors of “limited” overall study quality. Because of sample size, multivariate regression was restricted to two independent variables and performed using all pairs of significant univariate predictors. Variables with p < 0.05 in all paired multivariate regression analysis were reported as significant. SPSS Statistics (IBM. Armonk, NY) and STATA (Stata Corp, College Station, TX) were used for statistical analysis.

## Results

Forty-three pre-VA ablation CMR studies were performed between September 2015 and January 2020. Table 1. Shows the study demographics. One patient had a pacemaker (PM) and was not included in the ICD or non-ICD patient groups. The mean patient age was 59.8 ± 16.1 years, and 60% (n = 25) of the patients were males. The average LVEF was 0.47 ± 0.13. Over half of the subjects (57%) had an ICD. All ICDs were implanted in the left subclavicular region, except for one right sided implant. The ICD patient group had significantly more men, lower EF, greater NYHA class, and greater use of diuretics than the non-ICD group. Bh-LGE was not performed in four subjects without ICDs who received ss-SSFP-LGE due to arrhythmia or breath-holding difficulty. These subjects were classified as having limited motion quality bh-LGE studies but were excluded from contrast quality assessment. Bh-LGE was not performed in 4 subjects without ICDs who received ss-SSFP-LGE due to unrelated protocol development. These subjects were excluded from the bh-LGE quality analysis.

**Table 1:** Study demographics

	Total (n = 42)	Non-ICDs (n = 18)	ICDs/CRT-Ds (n = 24)	p-value
Age (years)	62.5 [51–69]	60 [41–69]	66 [52–71.5]	0.226
Males %	25 (60%)	7 (39%)	18 (75%)	0.027*
BMI (kg/m^2^)	29.6 [25.5–31.8]	27.85 [22.8–30.5]	30.45 [26.85–33.05]	0.131
**Etiology**				
ICM	11 (26%)	5 (28%)	6 (25%)	0.731
NICM	31 (74%)	13 (72%)	18 (75%)	0.731
ARVC	5 (12%)	2 (11%)	3 (13%)	1.000
Valvular heart disease	3 (7%)	2 (11%)	1 (4%)	0.567
HCM	2 (5%)	1 (6%)	1 (4%)	1.000
Sarcoid	2 (5%)	0 (0%)	2 (8%)	0.508
Idiopathic	19 (45%)	9 (50%)	10 (42%)	0.756
LVEF %	50 [35–60]	57 [50–60]	44.5 [30–52.5]	0.007*
**NYHA**				0.017*
Class I	23 (55%)	14 (78%)	9 (38%)	
Class I	15 (36%)	4 (22%)	11 (46%)	
Class III	4 (10%)	0	4 (16%)	
Class IV	0	0	0	
Diuretics	18 (43%)	2 (11%)	16 (67%)	0.001*
COPD	1 (2%)	1 (6%)	0	0.429
OSA	10 (24%)	3 (17%)	7 (29%)	0.473
Atrial fibrillation	13 (31%)	4 (22%)	9 (38%)	0.333

Continuous variables are reported as median and inter-quartile range. *ARVC/ARVD* Arrhythmogenic right ventricular cardiomyopathy, *BMI* Body mass index, *COPD* Chronic obstructive pulmonary disease, *HCM* Hypertrophic cardiomyopathy, *BMI* Body Mass Index, *LVEF* Left ventricular ejection fraction, *NICM* Non-ischemic cardiomyopathy, *NYHA* New York heart association, *OSA* Obstructive sleep apnea

(*) Statistically significant

### Cardiac and respiratory motion artifacts

**Fig. 2:**
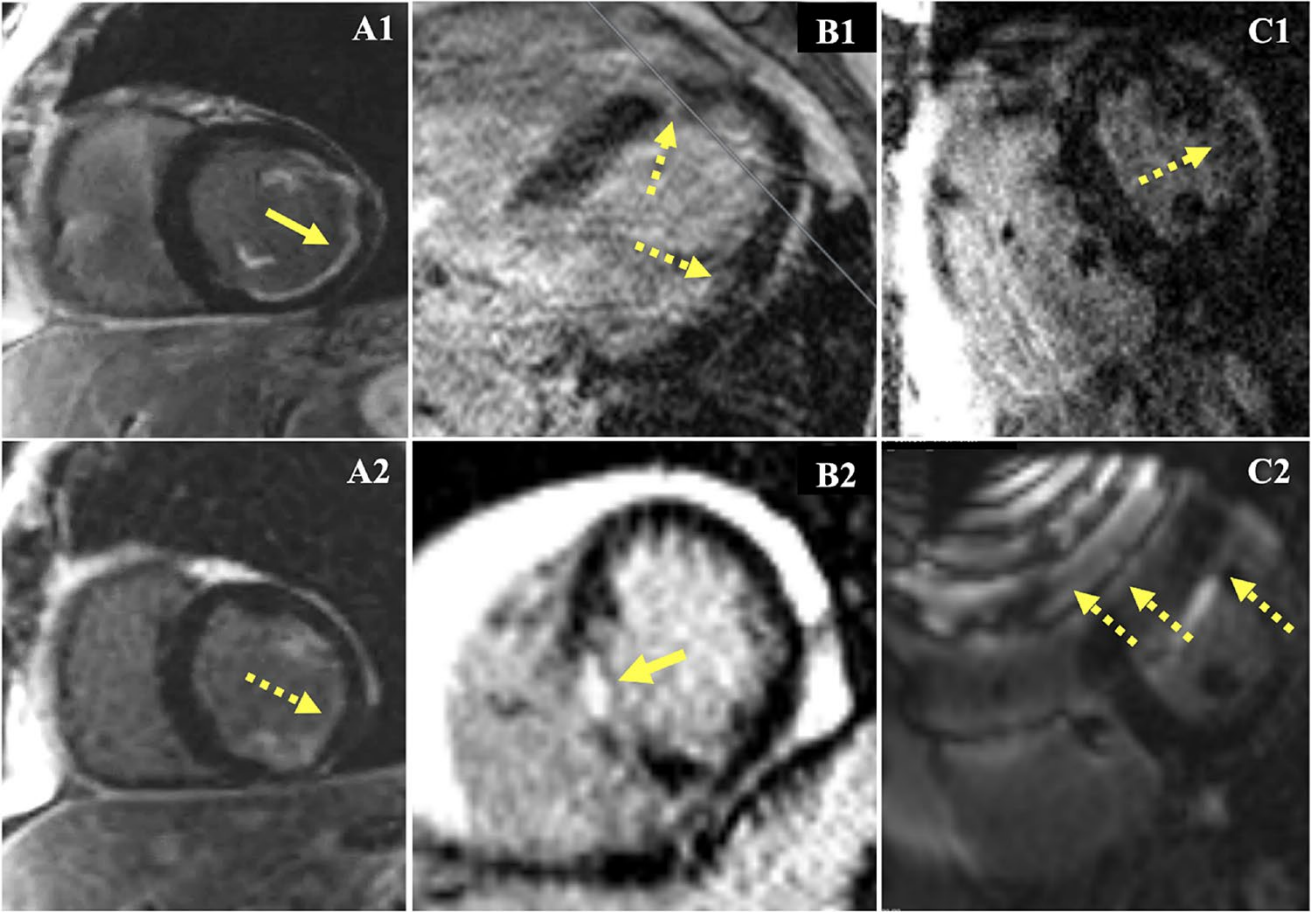
Effects of motion on pre-ablation LGE. **A1**, **2** Minimal motion artifact during LV short axis imaging. **A1** bh-LGE, and **A2** and ss-SSFP-LGE. Subtle features like subendocardial scar (solid yellow arrow) can be more difficult to detect on ss-SSFP-LGE images due to lower resolution and higher noise (dashed yellow arrow). **B** Motion in a patient without ICD. **B1** bh-LGE horizontal long axis image where respiratory motion limits distinction of scar from motion artifact (dashed yellow arrows). Short-axis imaging was not performed because of the subjects’ difficulty with breath-holding. **B2** Acceptable quality ss-SSFP-LGE short axis imaging could be performed in this subject. Unambiguous septal scarring is highlighted (yellow arrow). **C** Motion and noise affecting scar interpretation in a patient with an ICD. **C.1** Cardiac gated breath-hold image with limited interpretation of possible “patchy” scar (dashed yellow arrow). **C.2** ss-SSFP-LGE of the same short axis image slice is less helpful for reducing motion artifacts due to significant banding artifacts caused by ferromagnetic components in the ICD (dashed yellow arrows)

Examples of motion artifacts in single-shot SSFP and breath-held GRE LGE imaging are shown in Fig. 2. An “acceptable” motion study had a level of motion artifact that was not felt to limit scar interpretation on any short axis LGE image. In 7 of 18 non-ICD patients**,** single-shot imaging was selected over breath-held imaging because of reduced motion artifact (Table 2, see * comment). Non-ICD patients had significantly higher quality in terms of motion artifacts compared to ICD patients (94% vs. 46% acceptable motion studies; p = 0.001). In non-ICD patients, a median of no images per patient had interpretation limited by motion artifact. In ICD patients, motion limited interpretation of a median of 1.5 images per study and 5 images per motion artifact limited study. The Table 3, Motion limited section provides the number of patient studies and images limited by motion artifact.

**Table 2:** Limited quality single-shot SSFP vs. breath-hold GRE studies in non-ICD patients

	Non-ICD patients			
	ss-SSFP LGE	Breath-hold GRE LGE	p-value	Selected Study (ss-SSFP or bh-GRE LGE)
**Motion limited**				
#Studies classified	18	14	0.19	18 (11 ss-SSFP*, 7 GRE LGE**)
#Limited studies (%)	2/18 (11%)	5/14 (36%)		1/18 (5.6%)
**Contrast limited**				
#Studies classified	18	10	0.37	18 (11 ss-SSFP*, 7 GRE LGE**)
#Limited studies (%)	5/18 (28%)	1/10 (10%)		5/18 (28%)
**Overall limited**				
#Studies classified	18	14	0.46	18 (11 ss-SSFP*, 7 GRE LGE**)
#Limited studies (%)	5/18 (28%)	6/14 (43%)		5/18 (28%)

(*) ss-SSFP was selected because of improved motion quality in 7 of 11 cases. In the remaining 4 of 11 cases, ss-SSFP was selected because GRE LGE was not performed due to unrelated protocol development

(**) The breath-hold GRE LGE was selected if it had and equal or better quality than ss-SSFP LGE

**Table 3:** Limited quality studies and images in non-ICD vs. ICD patients

	Non-ICD patients (selected ss-SSFP LGE or bh-GRE LGE)	ICD patients wb-GRE LGE	p-value*
#Studies classified	18	24	
#Images classified	189	255	
Images/study	10.5 [9–12]	11 [10–12]	0.64
**Motion limited**			
#Limited studies (%)	1/18 (5.6%)	13/24 (54%)	0.001*
#Limited images (%)	4/189 (2.1%)	59/255 (23%)	< 0.0001*
#Limited images/study	0 [0–0]	1.5 [0–5]	0.0014*
#Limited images/motion limited study	4 [4–4]	5 [3–6]	1
**ICD limited**			
#Limited studies (%)	NA	6/24 (25%)	
#Limited images (%)	NA	33/255 (13%)	
#Limited images/study	NA	0 [0–1.5]	
#Limited images/ICD limited study	NA	5.5 [4–7]	
**Contrast limited**			
#Limited studies (%)	5/18 (28%)	9/24 (38%)	0.74
#Limited images (%)	13/189 (6.9%)	26/255 (10%)	0.24
#Limited images/study	0 [0–1]	0 [0–2]	0.48
#Limited images/contrast limited study	2 [1.8–3.5]	2 [2.0–4.2]	0.84
**Overall limited**			
#Limited studies (%)	5/18 (28%)	17/24 (71%)	0.012*
#Limited images (%)	17/189 (9.0%)	97/255 (38%)	< 0.0001*
#Limited images/study	0 [0–2]	5 [0–7]	0.0012*
#Limited images/overall limited study	3.0 [2–5]	6 [4.8–7]	0.032*

(*) Statistically significant

### ICD artifacts

**Fig. 3:**
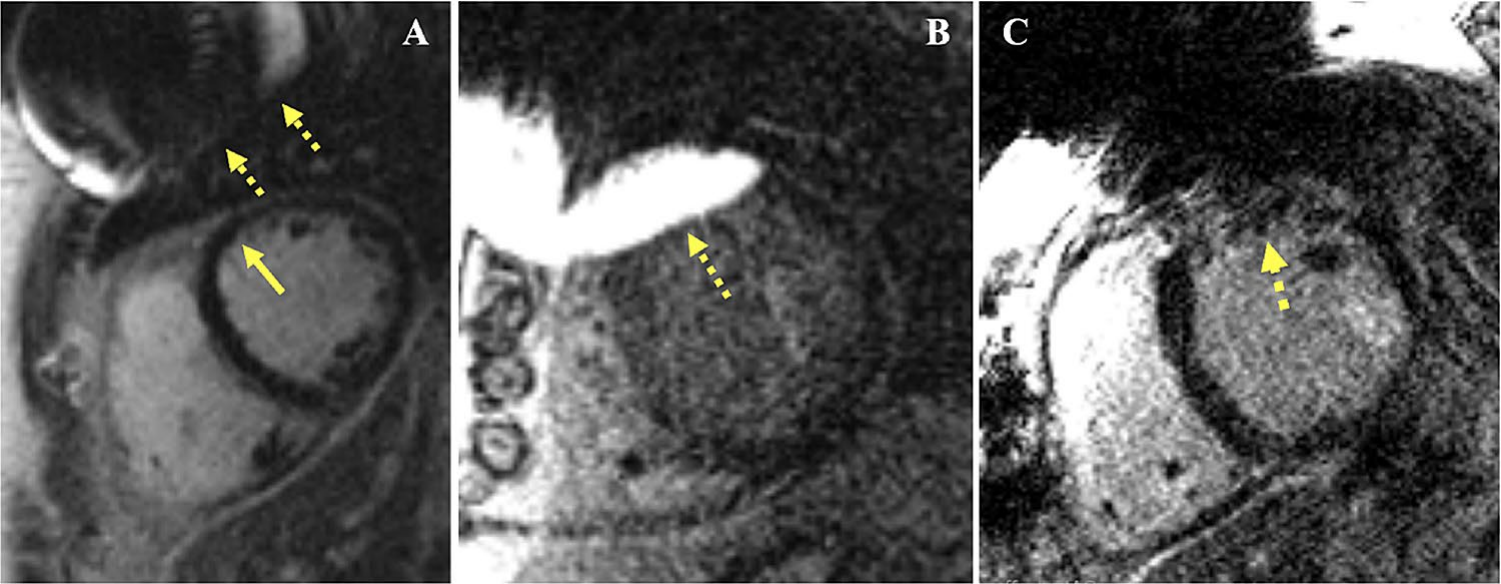
Effects of ICDs on pre-ablation LGE. **A** Acceptable ICD artifact (dashed yellow arrows), that is remote from the LV anterior wall (solid yellow arrow). **B**, **C** ICD artifacts limiting scar interpretation (dashed yellow arrows). **B** The LV anterior wall falls outside the bandwidth of the wide-bandwidth inversion RF pulse resulting in image hyperintensity. **C** The anterior wall is obscured by signal dropout and image distortion which occurs adjacent to the ICD

Examples of ICD artifacts in breath held LGE imaging are shown in Fig. 3. A study “limited” by ICD artifact had a level of artifact that limited scar interpretation on any short axis LGE image. Twenty-five percent of ICD patient studies were limited by ICD associated artifact. Hyper-intensity artifact overlying the heart was only seen in 2 patients (8%), with the remaining artifact due to ICD associated signal-dropout and image distortion artifacts. ICD artifacts predominantly affected the anterior wall of the left ventricle. Though ICD artifact limited a median of no image slices per ICD patient, for affected patients a median of 5.5 slices were limited**.** Single-shot SSFP imaging was not performed in ICD patients because of prohibitive ICD associated artifacts (Fig. 2C1). The Table 3, ICD limited section provides the number of patient studies and images limited by ICD artifact.

### Image contrast

An acceptable contrast study was defined as a level of scar to normal myocardial and blood pool contrast and noise that was not felt to limit scar interpretation on any short axis LGE image. For non-ICD patients, there was no significant difference in contrast quality between breath-hold studies compared to single-shot studies (90% vs. 72% acceptable contrast, p = 0.37). Similarly, there was no significant difference in contrast quality between patients without and with ICDs (72% vs. 62% acceptable contrast, p = 0.74). The Table 2, Contrast limited section provides the number of non-ICD patient studies that were limited by contrast quality. The Table 3, Contrast limited section compares the number of ICD and non-ICD patient studies and images that were limited by contrast quality.

### Overall study quality

**Fig. 4:**
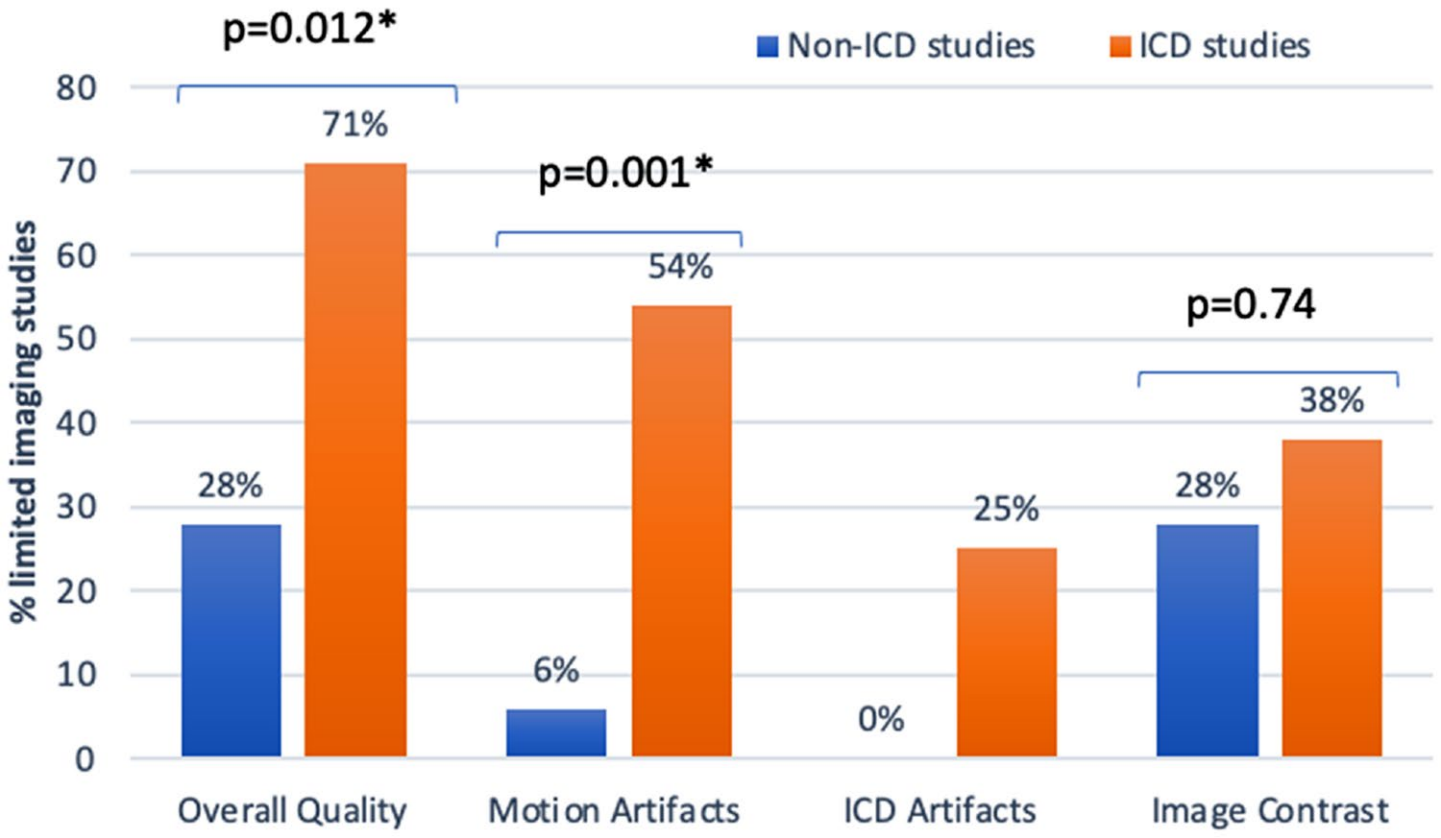
Contribution of motion artifact, ICD artifact, and contrast quality to imaging studies with limited whole-heart scar interpretation in ICD and non-ICD patients

Acceptable overall study quality was defined by a level of motion artifact, ICD artifact, and image contrast that was not felt to limit scar interpretation on any short-axis LGE image. Forty-eight percent of all pre-VA ablation CMR studies were scored as having acceptable quality. A significantly higher proportion of studies were acceptable for non-ICD patients as compared to ICD patients (72% vs 29%; p = 0.012) (Fig. 4). A median of 0 image slices per non-ICD patient had limited scar interpretation, compared to a 5 slices per ICD patient. Motion artifact, ICD artifact, and image contrast all contributed to suboptimal overall study quality in ICD patients (Fig. 4). The Table 3, Overall limited section provides the number of patient studies and images with overall limited quality.

Univariate demographic predictors of “limited” overall study quality were BMI > 30 kg/m^2^, presence of an ICD, LVEF ≤ 35%, and diuretic use (Table 4). Presence of an ICD was the only predictor that remained significant through multivariate regression of all pairs of univariate predictors with p < 0.15 (Supplementary Table 1).

**Table 4:** Predictors of limited overall study quality

Effect	“Limited” overall study quality (n = 22)	Univariate analysis		
		Odds ratio	95% CI	p-value
Age > 65 years	12 (54.5%)	2.23	0.64–7.74	0.207
Male	16 (72.7%)	3.26	0.09–11.80	0.072
BMI > 30 (kg/m^2^)	14 (63.6%)	4.08	1.12–14.86	0.033*
Presence of ICD	17 (77.3%)	7.93	1.99–31.59	0.003*
Atrial fibrillation	8 (36.3%)	1.71	0.45–6.51	0.428
LVEF ≤ 35%	10 (45.5%)	7.50	1.39–40.43	0.019*
NYHA class > I	13 (59.1%)	3.37	0.94–12.12	0.063
Diuretics	13 (59.1%)	4.33	1.16–16.25	0.03*
OSA	5 (22.7%)	0.88	0.21–3.65	0.863

COPD was not included in the regression model because only one patient had COPD

(*) Statistically significant

Study quality affected the agreement of scar presence/location between the study read and the official radiology read (Supplementary Table 2). Study quality was more important for assessing focal or heterogeneous “possible” scar (80% vs. 16.7% reader agreement for acceptable vs. limited quality studies, p = 0.00023). Disagreement in interpretation was due to the radiology reader under calling what the study reader felt was “possible” focal scar (7 studies), scar vs possible motion artifact (6 studies), basal heterogeneous/mid-wall scar (5 studies), and other sites of possible heterogeneous scar (1 study). Study quality related differences in reader agreement did not reach statistical significance when the study reader felt scar was clearly detectable above the image noise/artifact level (90% vs. 67% reader agreement for acceptable vs. limited quality studies, p = 0.11). Disagreement in interpretation of “clear” scar was due to the radiology reader under calling focal areas of scar (6 studies) and basal mid-wall scar (1 study). Four patients where ICD artifact rendered a significant portion of the LV uninterpretable were excluded from the reader comparison of scar presence/location.

### Individual image quality versus patient study quality

Quality scores improve when scar imaging quality is assessed for individual images rather than for the overall patient study (Table 3). For patients with ICDs, acceptable motion artifact was noted in only 46% of patients, but 77% of individual images. An acceptable level of ICD artifact was noted in 75% of patients compared to 87% of individual images. In patients without ICDs, acceptable overall quality was noted in 72% of studies compared to 91% of individual images. The conclusion that motion quality and overall quality was higher in patients without ICDs compared to those with ICDs remained significant for both overall study and individual image based quality assessment (p < 0.05, Table 3).

## Discussion

This study evaluated the reliability of contemporary CMR protocols for assessing myocardial scar prior to VA ablation. Image quality limited whole-LV scar assessment in more than half of ICD patients. This was mostly due to motion artifacts, though ICD artifacts and image contrast also contributed. In subjects without ICDs, motion-tolerant single-shot LGE imaging was effective at improving motion quality. Single-shot imaging was not performed in ICD patients because of significant ICD associated image artifacts using the conventional SSFP based method. Our results more clearly define the relative contributions of motion and ICD artifacts to suboptimal pre-VT ablation MRI quality as a guide to imaging protocol development. Imaging methods that improve motion quality are expected to be important for identifying not just gross scar location but also more specific anatomic features of scar that could serve as targets for ablation [4, 5].

### Motion considerations for pre-ablation myocardial scar assessment

Because of the limited speed of MRI, conventional LGE images are acquired across multiple heartbeats using cardiac gating to synchronize image data acquisitions to the same phase of the cardiac cycle [11]. To maintain the same position of the heart between beats, respiratory motion is frozen by breath-holding. Subjects with breath-holding difficulty and cardiac arrhythmia can have changes in the heart shape and location between heartbeats leading to motion artifacts [6, 11]. In this study, motion artifact for breath-hold imaging was predominantly due to respiratory motion. Frequent ventricular ectopy was present in some patients and in these cases also contributed to motion artifact. The prevalence of respiratory motion artifact suggests VA patients may have difficulty with breath-holding related to their comorbidities.

The ability of single shot imaging to suppress CMR motion artifact has been well described and current guidelines recommend its use in subjects with arrhythmia and breath-holding difficulty [6, 10]. Single-shot imaging collects all the information required to generate an image within a single heartbeat, reducing the impact of beat-to-beat variations cardiac and respiratory motion. However, image-resolution is limited by the need to collect all image information within a single heartbeat. Single-shot LGE is typically performed using rapid SSFP imaging to maximize the amount of image information that can be collected in a short time. However, SSFP is sensitive to distortion of the MR scanner magnetic field, such as the strong distortions created by ferromagnetic components of ICDs. The inability to perform single-shot SSFP imaging in ICD patients likely accounts for ICDs being an independent predictor of limited motion quality (Supplementary Table 3). Gradient recalled echo (GRE) imaging, typically used for cardiac-gated breath-hold LGE, is significantly less sensitive to ICD field distortion but is slower than SSFP. This results in lower single-shot image resolution, which appears to limit detection of arrhythmogenic scar features, such as scar border-zone and channels of viable tissue within scarred myocardium [12]. Contemporary motion-tolerant, multi-heartbeat CMR techniques such as respiratory motion gating, motion corrected averaging, and arrhythmia heartbeat-type gating will likely be useful for reducing motion artifact while preserving higher-resolution scar features during pre-ablation CMR [11, 13–15]. The interaction of motion and ICD artifacts needs to be considered during development of motion tolerant pre-VT ablation CMR since motion artifacts can be more pronounced in subjects with ICDs (Fig. 1A5) and motion tolerant, non-Cartesian MRI methods can exhibit significant blurring when magnetic field distortion is present, such as the strong distortion caused by ICDs.

### ICD artifact considerations for pre-ablation myocardial scar assessment

Prior to introduction of the wide-bandwidth inversion-recovery (wbIR) technique by Rashid and colleagues [9], the ability to perform LGE was significantly limited in ICD patients. wbIR was recently reported to permit scar interpretation in 15/16 (93%) cardiac segments [8]. We also found wbIR was effective at suppressing the hyperintensity artifact resulting from ICDs. However, other ICD artifacts like signal void artifacts and image distortion persist [8]. We found that 13% of wbIR images and 25% of wbIR patient studies had some ICD artifact overlapping part of the myocardium. Raising arms above the head appears helpful for shifting the ICD artifact further away from the heart [18]. This has not been systematically applied in our institution due to poor patient tolerance and was not performed in this study. Additional metal-artifact correction strategies could be evaluated for mitigating these artifacts, many of which have been developed for orthopedic MRI [16]. Performing a baseline CMR prior to ICD implantation would also avoid ICD related artifacts. However, a majority of these patient’s will not require VT ablation, and baseline imaging does not define changes in VT substrate that could occur between the time of ICD implant and VT ablation.

### Contrast considerations for pre-ablation myocardial scar assessment

Reliable detection of myocardial scar requires a sufficient contrast-to-noise ratio (CNR) to distinguish scar from noise, adjacent normal myocardium, and blood-pool. In this study, contrast quality was considered limited if the distinction of scar from noise was ambiguous and not attributable to motion or ICD artifacts. Some cases of ambiguous scar interpretation may have been due to “patchy” myocardial fibrosis which can be difficult to distinguish from noise at current LGE image resolution but may be detectable by statistical measures like entropy [17].

We found BMI was the only significant predictor of limited contrast quality (Supplementary Table 4. OR 15 (CI 2.72–82.67), p = 0.002). Higher BMI is expected to result in lower image signal relative to noise due to a greater distance of MRI receiver coils from the heart. ICDs subjects might also be expected to have reduce contrast quality because higher receiver bandwidth is often used to reduce ICD related image distortion but results in greater image noise (Fig. 1A4). However, contrast quality can also be reduced in non-ICD subjects because single-shot imaging is often used to suppress motion artifacts but requires a tradeoff of increased image noise or lower image resolution (Fig. 1B1). Respiratory gating methods can help address scar contrast and noise limitations by permitting signal averaging over longer imaging times that exceed a single breath hold [10, 13].

The determination of reduced contrast quality was potentially more subjective compared to the typical appearance of motion and ICD artifacts (Fig. 1). Supplementary Fig. 1 shows the effect of not considering contrast quality when determining overall study quality. For non-ICD patients, overall study quality improved from 72 to 94% acceptable studies. This was because the single-shot imaging consistently improved motion quality, but in some cases the resolution and noise tradeoff resulted in lower contrast quality scores. The number of limited quality ICD studies did not change because motion and ICD artifacts were also present.

### Comparison to prior studies

In our study, less than a half of wbIR LGE studies in ICD patients were reported to have adequate quality. This supports a recent report of pre-VT ablation MRI that found 3 of 10 patients studies were free of image artifact [15] but contrasts with another recent study of LGE imaging in ICD patients that reported 94% of image slices had overall adequate diagnostic quality [18]. This difference in part reflects our study’s focus on the quality of whole-LV scar assessment rather than the quality of individual images. We found that while only ~ 30% of ICD patients had overall acceptable study quality, a higher ~ 60% of individual image slices had adequate quality, and ~ 85% of image slices had adequate quality in terms of ICD related image artifacts.

Our evaluation of overall study quality rather than only individual image quality was felt justified for pre-ablation CMR because arrhythmogenic scar in any segment and transmural location of the heart can be used to guide invasive mapping and ablation. For example, epicardial scar has suggested a need for higher-risk epicardial access and mid-myocardial scar suggests need for an alternative to conventional RF ablation [3]. More detailed scar features like LGE border-zone corridors show promise for more specifically identifying favorable targets for ablation [5, 12, 19]. High-quality whole-heart scar assessment is also desirable for more automated scar segmentation which simplifies integration into clinical EAM workflows. This contrasts with conventional diagnostic CMR where uncertainty in a particular area of scar may be less important for grossly differentiating ischemic and various forms of non-ischemic cardiomyopathy (CM).

Residual differences in image quality from prior studies could be due to several factors. For example, Singh et.al. focused on reporting the clear benefits of wide-bandwidth inversion recovery in ICD patients [18]. The effects of demographic characteristics like obesity and whether imaging was performed prior to VA ablation were not evaluated. We found obesity had a significant adverse effect on contrast quality and was present in 73% of patients. All patients in this study were imaged prior to VA ablation, compared to 55% in that study. Differences in imaging technique and quality scoring, could also contribute to differences in image quality reported between studies.

Despite the quality limitations raised by this study, we believe the practice of performing pre-ablation CMR in subjects with ICDs remains reasonable since even incomplete information regarding scar distribution can be helpful for procedure guidance and given the current lack of alternative methods for transmural detection of myocardial scar. This study was meant to clarify which factors continue to impact study quality in VA ablation patients to help guide development of methods that address these issues. Limiting breath-hold number and duration to mitigate respiratory motion artifacts, arm raising to move ICD associated image artifacts further from the heart, and adjusting receiver bandwidth and image resolution to balance the tradeoff between image noise and ICD artifact may help to improve image quality until CMR methods that suppress both ICD and motion artifacts become more widely available.

## Study limitations

Limitations of this study include relatively small sample-size and retrospective evaluation of pre-ablation CMR referrals. These factors could lead to results that differ from the general VT ablation population. Although this study was performed at an experienced CMR center, protocol variations at other centers could lead to different results than those reported. CMR protocol variations that may have occurred over the course of this study were not accounted for. Gross changes in image quality and parameters known to affect ICD artifact, such as receiver bandwidth, were not noted between the start and end of the study. Second-reader validation was based on agreement rather than independent scoring, which could also bias results. Emerging CMR techniques to suppress motion artifacts, ICD artifacts, and improve contrast, are expected to improve image quality beyond what is reported [13, 16]. However, this study was aimed at assessing a widely available CMR protocol and to suggest which techniques may be most helpful for improving pre-ablation scar assessment. Although there is increasing interest in higher-resolution scar assessment using respiratory-gated 3D LGE, the quality of these images was not reported because of ongoing protocol development. The effect of image quality on the correlation of CMR scar to EAM scar requires further study but is also of interest, particularly for depicting the scar border-zone which is sensitive to image resolution and motion blurring [12, 20]. Prior studies have identified a number of challenges in correlating CMR to EAM including the limited transmural depth of EAM scar detection compared to transmural CMR scar detection, the contribution of fat to low voltage epicardial EAM, and the registration of modalities with different geometric depictions of the anatomy and different physical basis for scar detection [21, 22]. These considerations are particularly relevant in this study’s population where more than 70% of subjects had non-ischemic CM.

## Conclusion

In VA ablation patients with ICDs, conventional CMR protocols had a median of five image slices with limited scar interpretation due to motion, ICD artifact, or scar contrast, which limits whole-heart scar assessment. Motion artifacts contribute significantly to suboptimal image quality, particularly in patients with ICDs. Improved methods for motion and ICD artifact suppression will be helpful to reliably detect the high-resolution LGE scar features of interest for guiding VA ablation.

## Supplementary Information

Below is the link to the electronic supplementary material.

Supplementary file1 (DOCX 18 KB)

Supplementary file2 (DOCX 20 KB)
