# Supplementary material for: Quality assessment of cardiac magnetic resonance myocardial scar imaging prior to ventricular arrhythmia ablation
Source: Int J Cardiovasc Imaging. 2022 Nov 4;39(2):411–21. doi: 10.1007/s10554-022-02734-5 (PMC9870828; doi:10.1007/s10554-022-02734-5)
Supplement: Supplementary file 1 — Supplementary file1 (DOCX 18 KB) [file 10554_2022_2734_MOESM1_ESM.docx]

**Supplementary Figure 1.** Limited Quality Studies in non-ICD vs. ICD patients (excluding contrast quality from determination of overall study quality)
