# Supplementary material for: Quality assessment of cardiac magnetic resonance myocardial scar imaging prior to ventricular arrhythmia ablation
Source: Int J Cardiovasc Imaging. 2022 Nov 4;39(2):411–21. doi: 10.1007/s10554-022-02734-5 (PMC9870828; doi:10.1007/s10554-022-02734-5)
Supplement: Supplementary file 2 — Supplementary file2 (DOCX 20 KB) [file 10554_2022_2734_MOESM2_ESM.docx]

**Supplementary Table 1.** **Predictors of limited overall study quality**

===============================================================================================

Univariate regression: Overall limited study

-----------------------------------------------------------------------------------------------

limited studies (n=22) % Odds Ratio 95% CI p

______________________ ____ __________ _____________ _____

age_65_higher 12 54.5 2.23 0.64 7.74 0.207

male 16 72.7 3.26 0.9 11.8 0.072

**bmi_30_higher 14 63.6 4.08 1.12 14.86 0.033**

**presence_icd 17 77.3 7.93 1.99 31.59 0.003**

afib 8 36.4 1.71 0.45 6.51 0.428

**lvef_35_lower 10 45.5 7.5 1.39 40.43 0.019**

nyha_II_III 13 59.1 3.37 0.94 12.12 0.063

**diuretic 13 59.1 4.33 1.16 16.25 0.03**

osa 5 22.7 0.88 0.21 3.65 0.863

===============================================================================================

Multivariate regression (of each 2 of following variables with univariate regression P < 0.15):

-----------------------------------------------------------------------------------------------

limited studies (n=22) % Odds Ratio 95% CI p

______________________ ____ __________ _____________ _____

male 16 72.7 3.26 0.9 11.8 0.072

bmi_30_higher 14 63.6 4.08 1.12 14.86 0.033

presence_icd 17 77.3 7.93 1.99 31.59 0.003

lvef_35_lower 10 45.5 7.5 1.39 40.43 0.019

nyha_II_III 13 59.1 3.37 0.94 12.12 0.063

diuretic 13 59.1 4.33 1.16 16.25 0.03

-----------------------------------------------------------------------------------------------

Examples of first 3 of 15 regression analysis:

-----------------------------------------------------------------------------------------------

Multivariate regression 1 of 15 (of variables: age_65_higher, male): Overall limited study)

limited studies (n=22) % Odds Ratio 95% CI p

______________________ ____ __________ _____________ _____

male 16 72.7 2.44 0.63 9.53 0.198

bmi_30_higher 14 63.6 3.31 0.86 12.64 0.081

Multivariate regression 2 of 15 (of variables: age_65_higher, male): Overall limited study)

limited studies (n=22) % Odds Ratio 95% CI p

______________________ ____ __________ _____________ _____

male 16 72.7 1.66 0.37 7.4 0.504

presence_icd 17 77.3 6.58 1.51 28.61 0.012

Multivariate regression 3 of 15 (of variables: age_65_higher, male): Overall limited study)

limited studies (n=22) % Odds Ratio 95% CI p

______________________ ____ __________ ____________ _____

male 16 72.7 1.89 0.46 7.77 0.376

lvef_35_lower 10 45.5 5.74 0.97 34 0.054

===============================================================================================

Variables with P <= 0.05 in all 15 regression analysis (of every 2 variable grouping):

-----------------------------------------------------------------------------------------------

**presence_icd**

===============================================================================================

**Supplementary Table 2.** **Agreement of study read with official radiology read**

Acceptable Limited

Study Quality Study Quality p

________________ ________________ __________

radiology agreement clear scar (%) 18 / 20 (90.00%) 12 / 18 (66.67%) 0.11714

**radiology agreement possible scar (%) 16 / 20 (80.00%) 3 / 18 (16.67%) 0.00023377***

* Clear scar reflects scar that was clearly detected above the image noise/artifact level.

** Possible scar reflects focal or heterogenous scar that was less clearly detected above the image noise/artifact level.

*** The 2 disagreements of study reader assessed “clear scar” with the radiology read in acceptable quality images were due to focal scar.

**** The 6 disagreements of study reader assessed “clear scar” with the radiology read in limited quality images were due to focal scar (4 studies), and possible basal heterogeneous/mid-wall scar (2 studies).

**Supplementary Table 3.** **Predictors of unacceptable motion study quality**

===============================================================================================

Univariate regression: Motion limited study

-----------------------------------------------------------------------------------------------

limited studies (n=14) % Odds Ratio 95% CI p

______________________ ____ __________ ______________ _____

age_65_higher 9 64.3 3.24 0.85 12.36 0.085

male 11 78.6 3.67 0.84 16.04 0.084

bmi_30_higher 9 64.3 2.78 0.74 10.52 0.132

presence_icd 13 92.9 23.4 2.66 206.16 0.005

afib 6 42.9 2.25 0.58 8.77 0.243

lvef_35_lower 5 35.7 1.67 0.42 6.68 0.471

nyha_II_III 9 64.3 3.24 0.85 12.36 0.085

diuretic 9 64.3 3.8 0.98 14.67 0.053

osa 5 35.7 2.56 0.59 11 0.208

===============================================================================================

Multivariate regression (of each 2 of following variables with univariate regression P < 0.15):

-----------------------------------------------------------------------------------------------

limited studies (n=14) % Odds Ratio 95% CI p

______________________ ____ __________ ______________ _____

age_65_higher 9 64.3 3.24 0.85 12.36 0.085

male 11 78.6 3.67 0.84 16.04 0.084

bmi_30_higher 9 64.3 2.78 0.74 10.52 0.132

presence_icd 13 92.9 23.4 2.66 206.16 0.005

nyha_II_III 9 64.3 3.24 0.85 12.36 0.085

diuretic 9 64.3 3.8 0.98 14.67 0.053

-----------------------------------------------------------------------------------------------

Examples of first 3 of 15 regression analysis:

-----------------------------------------------------------------------------------------------

Multivariate regression 1 of 15 (of variables: age_65_higher, male): Motion limited study)

limited studies (n=14) % Odds Ratio 95% CI p

______________________ ____ __________ _____________ _____

age_65_higher 9 64.3 2.9 0.73 11.53 0.132

male 11 78.6 3.28 0.72 14.91 0.124

Multivariate regression 2 of 15 (of variables: age_65_higher, male): Motion limited study)

limited studies (n=14) % Odds Ratio 95% CI p

______________________ ____ __________ _____________ _____

age_65_higher 9 64.3 5.14 1.09 24.23 0.039

bmi_30_higher 9 64.3 4.56 0.96 21.59 0.056

Multivariate regression 3 of 15 (of variables: age_65_higher, male): Motion limited study)

limited studies (n=14) % Odds Ratio 95% CI p

______________________ ____ __________ ______________ _____

age_65_higher 9 64.3 2.3 0.48 10.96 0.294

presence_icd 13 92.9 20.61 2.3 184.67 0.007

===============================================================================================

Variables with P <= 0.05 in all 15 regression analysis (of every 2 variable grouping):

-----------------------------------------------------------------------------------------------

**presence_icd**

===============================================================================================

**Supplementary Table 4.** **Predictors of unacceptable contrast study quality**

===============================================================================================

Univariate regression: Contrast limited study

-----------------------------------------------------------------------------------------------

limited studies (n=14) % Odds Ratio 95% CI p

______________________ ____ __________ _____________ _____

age_65_higher 5 35.7 0.56 0.15 2.08 0.383

male 9 64.3 1.35 0.36 5.08 0.657

**bmi_30_higher 12 85.7 15 2.72 82.67 0.002**

presence_icd 9 64.3 1.8 0.48 6.74 0.383

afib 3 21.4 0.49 0.11 2.18 0.35

lvef_35_lower 5 35.7 1.67 0.42 6.68 0.471

nyha_II_III 7 50 1.33 0.37 4.83 0.661

diuretic 6 42.9 1 0.27 3.66 1

osa 2 14.3 0.42 0.08 2.3 0.315

===============================================================================================

Variables with P <= 0.05:

-----------------------------------------------------------------------------------------------

**bmi_30_higher**

===============================================================================================

**Supplementary Table 5 (Data for Supplementary Figure 1). Limited quality studies in non-ICD vs. ICD patients (excluding contrast quality from determination of overall study quality)**

**non_icd icd p**

**__________________ ____________________ __________**

**Motion limited studies # (%) {'1 / 18 (5.56%)'} {'13 / 24 (54.17%)'} 0.00098797**

**ICD limited studies # (%) {'0 / 18 (0.00%)'} {'6 / 24 (25.00%)' } 0.029197**

**Overall limited studies # (%) {'1 / 18 (5.56%)'} {'17 / 24 (70.83%)'} 2.2784e-05**
